# Supplementary material for: Why would individuals use indoor positioning systems? A study from a potential user interface perspective
Source: PLoS One. 2026 Mar 11;21(3):e0344133. doi: 10.1371/journal.pone.0344133 (PMC12978469; doi:10.1371/journal.pone.0344133)
Supplement: Attachment — Submitted filename: point-by-point response v10 sub.docx [file pone.0344133.s002.docx]

PONE-D-25-01491

**Why Would Individuals Use Indoor Positioning Systems? A Study from a Potential User Interface Perspective**

Point-by-Point-Response to the Reviewers

# **Introduction**

Dear Editor, dear Reviewers,

Thank you for the time and effort you invested in reviewing our manuscript. We appreciate your thoughtful comments and constructive suggestions, which helped us improve the clarity, rigor, and positioning of the paper. We have revised the manuscript accordingly and addressed each point below.

In the following, each comment is reproduced and answered in turn, with the corresponding changes indicated in the manuscript.

# 1 **Response to the Editor**

| ID: 1.1 | |
| --- | --- |
| Suggestion for improvement | Conceptual Validity: Prototype vs. Actual System (Crucial)  Both reviewers, particularly Reviewer 2, identify a fundamental disconnect between the study's claims and its stimulus.  The Issue: You evaluated a static/interactive User Interface (UI) mock-up, not a functional Indoor Positioning System (IPS). Real IPS usage involves signal latency, localization errors, and battery drain, none of which were experienced by users.  The Requirement: You must significantly qualify your findings. You cannot claim to measure "IPS adoption" in general. You are measuring the acceptance of a UI concept for indoor navigation. The manuscript must explicitly discuss this limitation and how the lack of technical friction (e.g., navigation errors) might inflate acceptance scores. |
| Answer | Thank you for highlighting this conceptual scope issue. We revised the manuscript to consistently frame the study as an early-stage evaluation of behavioral intention toward an interactive IPS UI mock‑up, rather than adoption of a fully functional IPS. The abstract, introduction, discussion, and limitations now explicitly state that the stimulus is a prototype UI concept and therefore does not include deployment-specific technical frictions (e.g., localization errors, latency), which may upward-bias intention. Accordingly, implications are formulated as prototype-based guidance (UI feature prioritization and value communication), not as evidence on real IPS adoption. See also responses to Reviewer 2 (IDs 3.1 and 3.2). |
| Change | see Abstract: added explicit scope qualification and prototype framing.  see Introduction: clarified that the study evaluates intention after task-based interaction with a UI mock‑up and bounded claims accordingly.  see Discussion / Conclusion (Limitations): emphasized intention-behavior gap and missing real deployment frictions. |

| ID: 1.2 | |
| --- | --- |
| Suggestion for improvement | Data Quality and Sampling Rigor  There are serious concerns regarding how the data was processed and the final sample size.  High Exclusion Rate: You excluded 268 out of 392 completed questionnaires (approx. 68% rejection rate). This is exceptionally high and suggests potential flaws in the survey design, task complexity, or attention checks. You must provide a detailed analysis of why so many participants failed.  Sample Size: The final sample (N=123) is considered small for a UTAUT2 model with moderators (Reviewer 1). You need to justify the statistical power of this sample size more robustly.  Demographics: The sample is skewed toward young German males/students. You must discuss how this limits generalizability to other populations (e.g., hospital visitors, elderly users). |
| Answer | We agree that the high exclusion rate and sampling implications require transparent reporting and justification. The numbers were indeed unclear. Thus, we expanded the Participants and Data Collection section to clearly describe recruitment via Clickworker, the predefined data-quality protocol (including code-based task completion and consistency checks), and the participant flow. In addition, we collected further data using the same protocol and pooled the independent data collections to increase the number of valid cases (final pooled sample: N = 181). Finally, we added limitations explicitly addressing potential selection effects due to strict screening and the resulting constraints on generalizability. See also Reviewer 2 ID 3.3 and Editor ID 1.3 regarding robustness/diagnostics. |
| Change | see Method 5.1 / 5.2: clarified task-based procedure and code-based attention checks.  see Method 5.3: expanded participant flow, exclusion logic, and sample description.  see Limitations: added paragraph on representativeness and selection effects from strict data-quality criteria.  see Appendix G: MANOVA Results. |

| ID: 1.3 | |
| --- | --- |
| Suggestion for improvement | Statistical Anomalies and Model Fit  The statistical results raise red flags regarding validity.  Suspiciously High R2: An explained variance of 0.859 is unusually high for behavioral intention studies (Reviewer 2). This suggests potential Common Method Bias (CMB), multicollinearity, or conceptual overlap between predictors. You must conduct and report rigorous tests for CMB and multicollinearity.  Insignificant Core Constructs: Many core UTAUT2 variables (Effort Expectancy, Facilitating Conditions, Hedonic Motivation, Habit) were non-significant. The discussion currently lacks a deep theoretical explanation for why these established predictors failed in this context.  Missing Data: Reviewer 1 notes that the specific list of items (especially for Spatial Abilities) and standard validity/reliability tables are missing or insufficient. These must be added. |
| Answer | Thank you for raising concerns about unusually high explained variance and potential statistical artefacts. We revised the reporting and added robustness diagnostics. The model now explains a substantial but more plausible share of intention variance (R² = 0.781) and focusses on the core constructs. We report (i) collinearity diagnostics for the structural model predictors (VIF < 3), and (ii) a common method assessment using Kock’s full-collinearity approach (full collinearity VIFs ranged 2.48–4.56; several exceed 3.3, but all are below 5). We also report SRMR and predictive assessments (PLSpredict, CVPAT) and interpret model fit indices cautiously. In the discussion and limitations, we explicitly bound interpretation to the prototype-based setting and discuss why some UTAUT2 constructs were not supported. See also Reviewer 2 ID 3.4 and ID 3.5. |
| Change | see Results: updated R² and structural paths; clarified inference approach (BCa bootstrap, one-tailed).  see Method 5.4 / Appendix B: added full-collinearity VIFs and additional diagnostic reporting (SRMR, PLSpredict, CVPAT).  see Discussion / Limitations: added interpretation of high R² and null findings as context-specific. |

| ID: 1.4 | |
| --- | --- |
| Suggestion for improvement | Theoretical Weaknesses: Spatial Abilities & Price Value  The theoretical additions to the model are viewed as weak or poorly operationalized.  Spatial Abilities (Hypothesis 8): Both reviewers criticize the inclusion of Spatial Abilities. It was found to be insignificant, yet the discussion does not adequately explain why. The operational definition of this construct is unclear, and its theoretical integration needs to be much stronger—or reconsidered.  Price Value: Setting a fixed hypothetical price (€0.99) is problematic (Reviewer 2). In a university context, students often expect free services. This framing likely skewed the results and needs to be discussed as a limitation. |
| Answer | We addressed both theoretical concerns. First, we removed Spatial Abilities and the associated hypotheses to keep the model aligned with canonical UTAUT2 and to avoid introducing a non-standard moderator without strong theoretical grounding. Second, we clarified that Price Value is conditional on the communicated pricing frame used in the study (one-time fee of €0.99) and therefore must be interpreted as a boundary condition rather than as a general pricing conclusion for IPS. The discussion and conclusion now explicitly state that alternative deployment models (institutional provision, subscriptions, free access) may alter price-related evaluations and should be tested in future research. See also Reviewer 1 ID 2.7 and Reviewer 2 ID 3.6. |
| Change | see Theory / Hypotheses / Research model: removed Spatial Abilities construct and all related hypotheses.  see Method 5.1: clarified Price Value measurement under the €0.99 one-time fee frame.  see Discussion / Conclusion: framed pricing implications cautiously as pricing-frame dependent. |

| ID: 1.5 | |
| --- | --- |
| Suggestion for improvement | Structure and Presentation  Introduction: Remove methodological details (sample size, specific stats) from the Introduction; focus on the theoretical gap.  Comparison: The literature review should better contrast outdoor vs. indoor navigation prototypes to justify the specific design choices made. |
| Answer | We revised the manuscript structure to improve clarity and theoretical positioning. The introduction now focuses on the research gap (behavioral intention toward IPS UI concepts) and states the study’s scope and contributions, while methodological detail is confined to the Method section. In addition, we added a dedicated subsection contrasting indoor vs. outdoor navigation acceptance research to clarify why OPS findings do not transfer directly to IPS UI contexts. Overall, the narrative now follows a clearer logic from (i) IPS context and use cases → (ii) UTAUT2-based model → (iii) task-based mock‑up evaluation → (iv) results and bounded implications. See also Reviewer 1 IDs 2.3 and 2.5. |
| Change | see Introduction: strengthened gap/contribution framing; reduced procedural detail.  see (new) Section 2.2: added OPS behavioral research contrast and transfer-limit argument. |

# 2 **Response to Review(er) 1**

| ID: 2.1 | |
| --- | --- |
| Suggestion for improvement | It is recommended to adhere to the academic writing style for a quantitative study, namely by refraining from utilising terms such as 'we' or 'our study' or ‘they’ in the text |
| Answer | We revised the manuscript to use a more formal academic style by reducing first-person phrasing and using neutral formulations (e.g., “this study”, passive voice) where appropriate. We also adjusted literature descriptions to avoid informal “they” phrasing and instead refer directly to authors/studies. |
| Change | All chapters: editing pass to reduce personal pronouns and informal wording. |

| ID: 2.2 | |
| --- | --- |
| Suggestion for improvement | Abstract \| The overall sample of 123 is insufficient for survey methods and does not align with the claim that IPS has gained popularity. The moderator should be highlighted in the abstract as a major contribution of this study. |
| Answer | Thank you for this helpful comment. We updated the abstract accordingly. The sample size is now reported as a pooled sample of N = 181 collected across independent data collections using the same protocol. We also revised the wording on IPS prevalence to avoid overstatement and instead note that real-world deployments remain comparatively scarce. Moderation (age, gender) is reported as a robustness analysis (UTAUT2). Most interaction effects were not supported because their BCa confidence intervals include zero. Only the Age × Price Value interaction showed a negative effect (see Appendix B, Table 19). Because adding moderators did not improve predictive performance, moderation it was removed from the main analysis as suggested by the AE. Thus, is documented as robustness evidence rather than positioned as the main contribution in the abstract. |
| Change | see Abstract: updated sample size (N=181), adjusted IPS deployment wording, and clarified scope.  see Method / Appendix: moderation robustness analysis documented. |

| ID: 2.3 | |
| --- | --- |
| Suggestion for improvement | Introduction \| The research problem appears insufficiently robust to warrant the application of UTAUT2. Researchers employ UTAUT to understand the factors that influence both intentions and actual behaviour when using technology. However, the problem articulated primarily concentrated on technological or system design concerns.  The content should be realigned with the sub-title; for instance, in the Introduction section, the sample size and statistical methods should not be reiterated, as they are already covered in the abstract and subsequently in the methodology section. The study's significance focusses primarily on application, neglecting theoretical contributions. |
| Answer | We revised the introduction to strengthen the research problem and theoretical contribution. The introduction now emphasizes the gap that IPS research often focuses on technical feasibility, while evidence on adoption drivers and intention formation grounded in UI interaction remains limited. We also state the study’s contributions (use-case-driven UI mock‑up + task-based evaluation + UTAUT2 determinants) and keep methodological specifics (sample size, analysis technique) in the Method section. The research questions are now explicitly stated. |
| Change | see Introduction: rewritten gap statement, contributions, and explicit research questions; removed methodological detail from the introduction. |

| ID: 2.4 | |
| --- | --- |
| Suggestion for improvement | Literature Review – Indoor Positioning Systems \| The discussion primarily emphasises that previous studies fail to address the aspect of intention to use. Given that the technology purports to possess a distinctive system, a robust rationale must be articulated regarding the necessity of employing the theory in relation to this unique system. |
| Answer | We expanded the theoretical background to better cover IPS-related research and to justify the choice of UTAUT2 for evaluating intention toward an IPS UI concept. The revised background now integrates IPS use cases and UI prototyping literature and positions UTAUT2 as suitable for voluntary, consumer-like, early-stage adoption evaluations. |
| Change | see Sections 2–4: expanded IPS/UI background and LBS adoption evidence; clarified rationale for UTAUT2. |

| ID: 2.5 | |
| --- | --- |
| Suggestion for improvement | Literature review – Developing an IPS prototype \| Since the author(s) have noted the differences in navigation outdoors. This section must compare the two prototypes to provide a clear description. |
| Answer | We addressed this by adding a dedicated subsection on outdoor navigation (OPS) behavioral research and explicitly contrasting OPS vs. IPS in constraints, technologies, and typical use cases. This section clarifies why OPS acceptance findings cannot be transferred directly to IPS UI concepts and motivates the need for IPS-specific intention research. |
| Change | see Section 2.2: added OPS acceptance studies and an explicit indoor-outdoor contrast argument. |

| ID: 2.6 | |
| --- | --- |
| Suggestion for improvement | Literature review – theory for evaluation \| Similar to the previous discussion, it indicated that the theory has not been tested in the IPS prototype. No novel components have been incorporated into the theory, owing to the distinctive prototype of the technology discussed in prior sections. The requirements of the moderator within the theory should be emphasised in the discourse, along with the rationale for including spatial abilities within the theory. The points may be incorporated in the introduction as research issues. |
| Answer | We revised the model to align with UTAUT2 and added the standard moderators age and gender as a robustness analysis (direct effects and interactions with all predictors). We report the moderation model output and compare model performance with the main model in the Appendix. In addition, the previously included Spatial Abilities construct was removed (see ID 2.7). |
| Change | see Method 5.4 / Appendix B: added moderation robustness analysis (age, gender) and performance comparison.  see Research model: Spatial Abilities removed. |

| ID: 2.7 | |
| --- | --- |
| Suggestion for improvement | Literature Review – Hypotheses & Research Model \| The discussion appears to have led to a superficial hypothesis. Given the significance of Hypothesis 8 to the theory, the debate necessitates the strengthening and enhancement of Hypothesis 8. There is a lack of comprehensive reviews on topics related to spatial ability. |
| Answer | We agree and removed the Spatial Abilities construct and all associated hypotheses (formerly H8.*). This keeps the model consistent with UTAUT2 and avoids introducing a moderator without strong theoretical grounding in this context. |
| Change | see Hypotheses / Research model / Results: removed Spatial Abilities and all related hypotheses and reporting. |

# 3 **Response to Review(er) 2**

| ID: 3.1 | |
| --- | --- |
| Suggestion for improvement | First, a central limitation of the study is that it does not evaluate an actual indoor positioning system but rather an interactive user interface prototype without real positioning technology. Critical characteristics of IPS—such as localization accuracy, signal instability, latency, or navigation errors—are not part of the user experience. Nevertheless, the manuscript repeatedly draws conclusions about the adoption and use of “IPS” in general. This conceptual leap risks overstating the implications of the findings and should be substantially qualified. |
| Answer | Thank you. We revised the manuscript to avoid conceptual overreach and to clearly communicate what the data can and cannot support. The manuscript now consistently frames the study as measuring behavioral intention toward an interactive IPS UI mock‑up concept after task-based interaction, not adoption of a fully deployed IPS. We explicitly note the absence of deployment-specific frictions (e.g., localization inaccuracies, latency) and bound implications accordingly. See also Editor ID 1.1 and Reviewer 2 ID 3.2. |
| Change | see Abstract / Introduction / Discussion / Limitations: added explicit prototype framing and bounded interpretation. |

| ID: 3.2 | |
| --- | --- |
| Suggestion for improvement | Second, although the authors attempt to reduce the intention–behavior gap by requiring interaction with the prototype, the study still relies exclusively on self-reported behavioral intention in an artificial and short-term setting. Participants complete predefined tasks in a mock-up environment under monetary incentives, which does not reflect real-world usage, sustained adoption, or behavioral consequences. As a result, the external and ecological validity of the findings remains limited. |
| Answer | We agree that intention does not equal usage behavior. We therefore strengthened the discussion and limitations to explicitly address the intention–behavior gap. We clarify that the task-based mock‑up interaction increases concreteness compared to a purely abstract scenario but still measures self-reported intention. We also outline future research using field deployments, longitudinal designs, and behavioral log data to assess actual usage and post-adoption dynamics. |
| Change | see Discussion / Limitations: added explicit intention-behavior limitation and concrete future research directions (field/longitudinal/behavioral data). |

| ID: 3.3 | |
| --- | --- |
| Suggestion for improvement | Third, the sampling strategy raises concerns. Of the 392 completed questionnaires, 268 were excluded due to failed control questions, resulting in a final sample of only 123 participants. This exceptionally high exclusion rate warrants a more critical discussion, as it may indicate issues related to task complexity, participant engagement, or survey design. Furthermore, the sample is heavily skewed toward young, male, German university members, which severely limits generalizability to other user groups, cultural contexts, and indoor environments such as hospitals or airports. |
| Answer | We expanded the Method section to provide a transparent account of sampling and data quality. We now report recruitment via Clickworker, compensation, and the strict predefined data-quality criteria (including code-based task completion and additional checks). The participant flow is described, and we discuss the implication that strict screening can lead to high exclusion rates in compensated crowdworking settings. We also added limitations on representativeness and generalizability and note that replications with more diverse samples and settings are needed. See also Editor ID 1.2. |
| Change | see Method 5.3: expanded participant flow and exclusion criteria reporting.  see Limitations: added selection effects/generalizability discussion. |

| ID: 3.4 | |
| --- | --- |
| Suggestion for improvement | Fourth, the reported explained variance of behavioral intention (R² = 0.859) is unusually high for technology acceptance research. Such a value may point to conceptual overlap between predictors, common method bias, or an overly context-specific model. The manuscript does not sufficiently reflect on this issue, nor does it discuss potential inflation of explanatory power. |
| Answer | We addressed the concern about unusually high explained variance by strengthening diagnostic reporting and interpretation. The revised model explains R² = 0.781, and we report collinearity diagnostics (predictor VIFs < 3). In addition, we conducted Kock’s full-collinearity assessment for potential common method bias (full collinearity VIF range 2.48–4.56). We interpret the high R² cautiously as potentially amplified by (i) a homogeneous context, (ii) belief overlap, and (iii) single-method measurement, and we explicitly bound conclusions to the prototype-based evaluation setting. See also Editor ID 1.3. |
| Change | see Method 5.4 / Appendix B: added full-collinearity assessment and diagnostic reporting.  see Discussion / Limitations: added cautious interpretation of high R² and bounded generalization. |

| ID: 3.5 | |
| --- | --- |
| Suggestion for improvement | Fifth, several core UTAUT2 constructs—effort expectancy, facilitating conditions, hedonic motivation, and habit—do not show significant effects. While null findings are reported, their theoretical implications are not deeply explored. Similarly, the inclusion of spatial abilities as a moderating variable appears weakly justified and empirically unsupported, raising questions about its conceptual integration within the model. |
| Answer | We expanded the discussion of non-supported hypotheses. Effort Expectancy, Facilitating Conditions, and Hedonic Motivation were not supported because their one-tailed BCa confidence intervals include zero. We interpret these results as context-dependent and potentially affected by ceiling effects (ease of use), the lack of real infrastructure constraints in a mock‑up, and the utilitarian nature of indoor navigation. We also interpret Habit carefully: in a mock‑up study it likely reflects a predisposition toward routinized navigation-tool use rather than true habit formation with the specific system. Finally, the previously included Spatial Ability construct was removed (see Reviewer 1 ID 2.7). |
| Change | see Discussion: added structured explanation of null effects and careful interpretation of Habit in a UI prototype context.  see Research model (Figure 2): removed Spatial Ability. |

| ID: 3.6 | |
| --- | --- |
| Suggestion for improvement | Finally, the operationalization of price value through a fixed hypothetical price (€0.99) introduces a strong framing effect. This price does not reflect realistic IPS deployment models (e.g., institutional provision, subscription-based services, or free access), limiting the interpretability and transferability of the observed price value effect. |
| Answer | We clarified that Price Value was measured under a specific communicated pricing scheme (one-time fee of €0.99) and therefore reflects a pricing-frame dependent benefit–cost assessment. We now explicitly treat Price Value as a boundary condition and caution against generalizing to other deployment models (e.g., institutional provision, subscriptions, free access). We also added future research directions recommending experimental variation of pricing models and levels. See also Editor ID 1.4. |
| Change | see Method 5.1: clarified Price Value operationalization under the €0.99 one-time fee frame.  see Discussion / Conclusion: bounded pricing implications and added future research suggestions. |
